# Integrative Metabolomic Characterization Reveals the Mediating Effect of *Bifidobacterium breve* on Amino Acid Metabolism in a Mouse Model of Alzheimer’s Disease

**DOI:** 10.3390/nu14040735

**Published:** 2022-02-09

**Authors:** Guangsu Zhu, Min Guo, Jianxin Zhao, Hao Zhang, Gang Wang, Wei Chen

**Affiliations:** 1 State Key Laboratory of Food Science and Technology, Jiangnan University, Wuxi 214122, China; su1994112@163.com (G.Z.); guomin@jiangnan.edu.cn (M.G.); zhaojianxin@jiangnan.edu.cn (J.Z.); zhanghao@jiangnan.edu.cn (H.Z.); wanggang@jiangnan.edu.cn (G.W.); 2 School of Food Science and Technology, Jiangnan University, Wuxi 214122, China; 3 (Yangzhou) Institute of Food Biotechnology, Jiangnan University, Yangzhou 225004, China; 4 National Engineering Center of Functional Food, Jiangnan University, Wuxi 214122, China; 5 Wuxi Translational Medicine Research Center, Jiangsu Translational Medicine Research Institute Wuxi Branch, Wuxi 214122, China

**Keywords:** metabolomics, Alzheimer’s disease, *Bifidobacterium*, amino acid metabolism

## Abstract

Alzheimer’s disease (AD) is commonly accompanied by global alterations in metabolic profiles, resulting in cognitive impairment and neuroinflammation in the brain. Using ultraperformance liquid chromatography-mass spectrometry, we performed integrative untargeted metabolomic analysis of metabolite alterations in the serum and hippocampal tissues of amyloid-β (Aβ)-injected AD model mice and sham controls. Multivariate analysis revealed that a *Bifidobacterium breve* CCFM1025 intervention significantly restored the differential metabolites induced by Aβ-injection, resulting in *B. breve* CCFM1025 serum and hippocampal metabolomes clustering between control and model mice. Furthermore, pathway and metabolite set enrichment analysis found that these altered metabolites were predominantly linked to amino acid metabolism. Overall, the integrative metabolome analysis indicated that *B. breve* CCFM1025 supplementation could modulate serum and hippocampal metabolomes in the early stage of AD, with amino acids as a potential driver.

## 1. Introduction

Alzheimer’s disease (AD) is a neurodegenerative disease predominantly seen in elderly individuals (mostly older than 60 years). This disease is prevalent worldwide and characterized by the deposition of amyloid plaques, neuronal dysfunction, and behavior defection, which increase the health care burden on society [1]. Patients suffering from AD undergo a progressively complex condition: initial preclinical AD progresses to mild cognitive impairment and eventually develops into dementia [2]. Although this gradual progression limits the complete understanding of the disease’s complex pathology, it also creates an opportunity for disease-modifying interventions in the early stage of AD [3].

In the past decade, microbiota-targeted interventions have been proposed as a promising therapeutic approach for neurological disorders, including AD and Parkinson’s disease. Psychobiotics are known for their ability to confer positive psychiatric effects through interactions with commensal gut bacteria, when ingested in appropriate quantities. Studies have proposed roles for *Lactobacillus rhamnosus* GG [4], *Bifidobacterium breve* A1 [5], and *B. breve* CCFM1025 [6] in alleviating the symptoms of AD, but exactly how these psychobiotics confer beneficial effects on behavior and brain function remains unclear.

Metabolomics uses high-throughput analytical techniques to identify small molecules in biological samples and tissues. The metabolomic platform is commonly based on mass spectrometry and equipped with a liquid chromatography separation system. When applied in an untargeted manner, this approach incorporates the largest spectral range and compares the relative abundances of all metabolites in multiple samples [7]. Compared with genomic or proteomic signatures, metabolic alterations represent molecular biology and can be used to directly observe different disease stages [8]. Therefore, untargeted metabolomics is a promising tool to identify potential biomarkers and explore pathways.

Generally, untargeted metabolomics has been performed on biological and tissues, including serum and/or plasma, urine, hippocampus, liver, and fecal samples [9]. In animal models, elevated levels of the metabolite arginine in the brain and alterations in serum concentrations of glutamine and proline have been described in APP/PS1 mice compared with wild-type controls [10]. Moreover, an earlier study demonstrated that alterations in phospholipids and amino acids in the hippocampus also led to a memory deficit in an AD mouse model [11]. In human studies, alterations in amino acid metabolites, notably arginine and N-acetylaspartate, and reductions in linoleic acid were observed in AD individuals compared with healthy controls [12]. Furthermore, Guiraud et al. [13] reported significant differences in the levels of glycine, S-adenosylmethionine, and S-adenosylhomocysteine between cerebrospinal fluid samples from AD individuals and healthy controls. Although these studies were heterogeneous—using different metabolomics approaches and performed under different experimental settings, with different animal models and samples and different subtypes and stages of AD—they deciphered several common metabolite alterations in the progression of AD.

Our previous study demonstrated that *B. breve* CCFM1025 alleviated cognitive impairment and demonstrated beneficial effects on brain function in an AD mouse model [6], with a potential psychobiotic effect in delaying the progression of AD. This study aimed to identify metabolic alterations that are potentially involved in the development of amyloid-β (Aβ)-injected behavior defection and *B. breve* CCFM1025 intervention. To this end, we performed an untargeted metabolomic analysis of serum and hippocampus samples. Herein, we report that the metabolites differed between control and model mice, both in serum and hippocampus samples, whereas the metabolites in *B. breve* CCFM1025-treated mice were more similar to the control mice. Furthermore, we indicated that *B. breve* CCFM1025 administration restored aspects of the serum and hippocampal metabolomes of Aβ-injected mice to attenuate cognitive impairment via modulation of the amino acids.

## 2. Materials and Methods

### 2.1. Bacterial Treatment

*B. breve* CCFM1025 was isolated from fecal samples of a healthy human, with written informed consent obtained to use those fecal samples for research purposes. *B. breve* CCFM1025 was freshly cultured at 37 °C under anaerobic conditions. For oral administration, the bacteria cells were washed and re-suspended in 10% skimmed milk to a final concentration of 5 × 10^9^ colony-forming units/mL.

### 2.2. Animals

Eight-week-old male adult C57BL/6J mice were purchased from the Model Animal Research Center of Nanjing University (Nanjing, China). All experiments were approved by the Animal Experimentation Ethics Committee of Jiangnan University (JN.No20190415c0800618(74)). Animals were kept under a 12-h light-dark cycle at 22 °C ± 3 °C and humidity of 55 ± 10%. Standard food and sterile water were given ad libitum.

After acclimatization for 1 week, mice were assigned to 3 experimental groups (*n* = 8 per group): control, model, and CCFM1025. To establish an AD animal model, mice in the model and CCFM1025 groups received an intrahippocampal injection of 1 μL of Aβ1-42 oligomer [6]. Mice were gavaged once a day for 6 weeks with 200 μL of bacterial suspension (CCFM1025 group) or sterile 10% skimmed milk as a vehicle control (control and model groups).

### 2.3. Serum and Hippocampus Collection

At the end of the experiment, the mice were deeply anesthetized, and blood samples were obtained. Subsequently, the mice were decapitated, and their brains were isolated. The left hippocampus was carefully dissected and immediately stored at −80 °C for metabolomic analysis. For serum sample collection, blood samples were left to clot for 2 h at room temperature and then centrifuged at 3500× *g* at 4 °C for 15 min. Serum sample aliquots were stored at −80 °C until analysis.

### 2.4. Metabolomics

#### 2.4.1. Metabolite Sample Preparation

Metabolites in serum were extracted as detailed in Yuan et al. [14]. Aliquoted serum samples stored at −80 °C were thawed and centrifuged at 14,000× *g* for 5 min. For metabolite extraction, 300 µL of 80% (vol/vol) methanol (pre-cooled to −80 °C) were added to 100 µL of supernatant. The extraction solvent was mixed by vortexing at a maximum speed for 2 min and then centrifuged at 4 °C at 14,000× *g* for 15 min, after which the supernatant was transferred to a new microcentrifuge tube. Samples were concentrated using a SpeedVac system (Thermo Fisher Scientific, MA, USA) and vacuum dried. For liquid chromatography with tandem mass spectrometry (LC-MS/MS) analysis, samples were re-suspended in 80% (vol/vol) methanol and transferred to glass vials.

Metabolites in the hippocampal tissues were extracted as detailed in Olson et al. [15]. Briefly, hippocampal tissues were homogenized in 1 mL of 80% (vol/vol) methanol (pre-cooled to −80 °C) and vigorously mixed on dry ice for 1 min. After refrigerated centrifugation (14,000× *g*, 15 min), the resulting supernatant was transferred into a new microfuge tube, concentrated, and dried under vacuum. Before analysis, dried samples were reconstituted in 70% acetonitrile (vol/vol) and transferred to glass autosampler vials. For quality control (QC) purposes, a QC sample was prepared by pooling 10 µL of every sample.

#### 2.4.2. Ultraperformance Liquid Chromatography-Mass Spectrometry (UPLC-MS) Analysis Parameters

Samples were analyzed using an ItiMateU-3000 ultraperformance liquid-chromatography (UPLC) system (Thermo Fisher Scientific, MA, USA) coupled to a high-resolution Q-Exactive mass spectrometer (Thermo Fisher Scientific, MA, USA). A Waters Acquity UPLC T3 column (2.1 × 100 mm, 1.8 µm) was used at an operating temperature of 30 °C.

The mobile phases were A) 0.1% formic acid in negative mode or 5 mM ammonium acetate in negative mode and B) 100% acetonitrile. The analytical gradient was: 0–1 min, 5% B; 10 min, 99% B; 12 min, 99% B; 3.5 min, 95% B; 12.1 min, 5% B; 15 min, 5% B. The flow rate was 0.3 mL/min with an injection volume of 2 µL for both phases. Samples were held at 4 °C in the autosampler.

The Q Exactive was run with polarity switching (+3.80 kV/−3.20 kV) in full scan mode with an *m*/*z* range of 70–1050. The electrospray ionization (ESI) source conditions were set as follows: sheath gas flow of 40 psi, aus gas flow of 10 psi, capillary temperature of 320 °C, and aus gas heater temperature of 350 °C. The normalized collision energy (NCE) was set to 20–40–60 eV.

#### 2.4.3. Metabolomic Data Analysis

Mass raw data files were imported into Compound Discovery 3.1 (Thermo Fisher Scientific, MA, USA) where peaks were automatically processed using an untargeted metabolic workflow. The processing workflow used in this study is provided in Supplementary Figure S1. Metabolites were identified by matching the Human Metabolome Database (HMDB), Kyoto Encyclopedia of Genes and Genomes (KEGG), mzCloud, and ChemSpider databases. The resulting data matrix was imported into an Excel file. Following log transformation and pareto scaling for each compound, data were further analyzed using SIMCA 14.1 software (Umetrics, Umea, Sweden).

Based on the relative quantification of metabolites, principal component analysis (PCA) of all samples and the Pearson correlation coefficient between QC samples were set as standards in the assessment of stability in the metabolomic data sets [16]. Partial least squares discriminant analysis (PLS-DA) and orthogonal partial least squares discriminant analysis (OPLS-DA) were constructed to identify discriminatory features in relevant comparisons. The number of permutation tests was set to 200. Differential metabolites were identified based on a variable important in the projection (VIP) > 1, *p* < 0.05 and fold change (FC) > 1.5 or < 0.67 [17]. Hierarchical cluster analysis, pathway analysis, and metabolite set enrichment analysis (MSEA) were performed in MetaboAnalyst 5.0 (https://www.metaboanalyst.ca/, accessed on 8 December 2021).

## 3. Results

### 3.1. Quality Assessment and Annotated Metabolites of the Overall Metabolome

**Figure 1:**
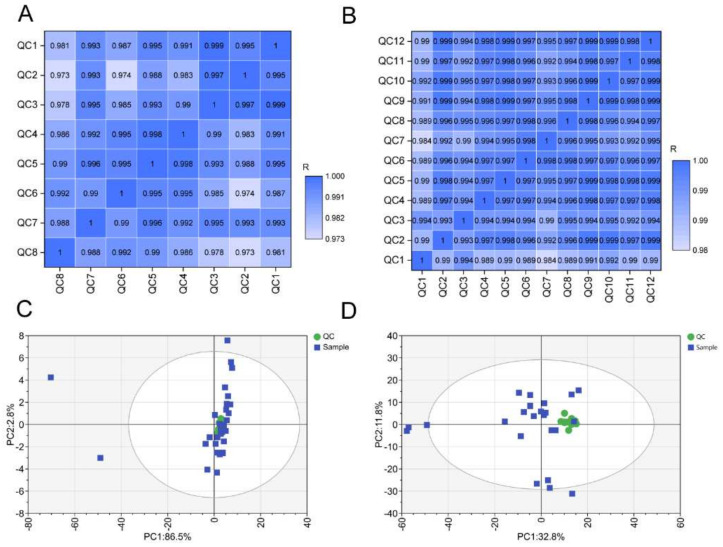
Quality control assessment. The Pearson correlation of serum (**A**) and hippocampus tissues (**B**) in ESI^−^ mode. The PCA score plots for all serum (**C**) and hippocampus (**D**) samples containing QC samples in ESI^−^ mode. ESI^−^: negative electrospray ionization.

To assess the stability of the metabolomic profiles, we first performed Pearson correlation analysis of the QC samples. As shown in the correlation plot, the Pearson correlation coefficients of both serum and hippocampal negative electrospray ionization (ESI^−^) QC samples were high (Figure 1A,B). Similar results were also observed in serum and hippocampal positive ESI (ESI^+^) QC samples (Supplementary Figure S2A,B). Moreover, the serum and hippocampal QC samples in both ESI^−^ (Figure 1C,D) and ESI^+^ (Supplementary Figure S2C,D) modes clustered together in the PCA plots.

**Figure 2:**
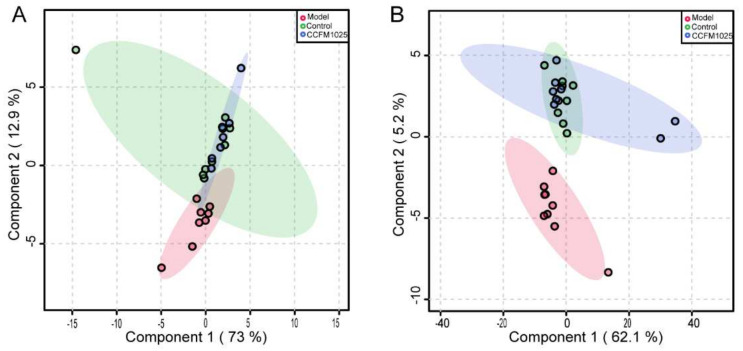
PLS-DA score plot for serum (**A**) and hippocampus (**B**) samples among all groups. PLS-DA: partial least squares discrimination analysis.

By matching online databases, 2198 metabolites were detected in all samples, including 302 ESI^−^ (235 with defined names) and 347 ESI^+^ (221 with defined names) serum metabolites, and 301 ESI^−^ (205 with defined names) and 1248 ESI^+^ (866 with defined names) hippocampal metabolites. A PLS-DA analysis based on these features indicated that the *B. breve* CCFM1025 group was more similar to the control group and clearly separated from the model group (Figure 2A,B).

### 3.2. B. breve CCFM1025 Supplementation Modulated the Aβ-Induced Serum Metabolome

**Figure 3:**
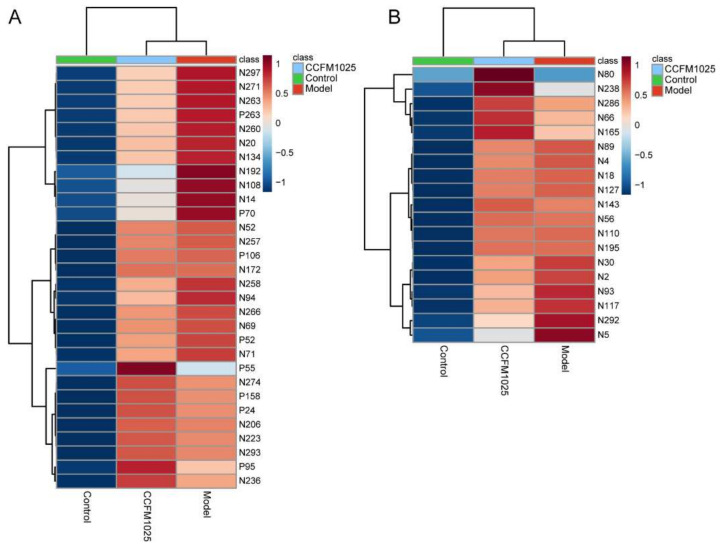
Hierarchical cluster analysis of differential metabolites among the control, model, and *B. breve* CCFM1025 groups in serum (**A**) and hippocampus (**B**) samples, respectively. Among-group comparisons were performed using one-way ANOVA. The name of differential metabolites was listed in Supplementary Tables S1 and S2.

Given the Aβ-induced changes in the serum and effects on hippocampal function, we first evaluated the relative changes in serum metabolites among the three groups. Compared with the control mice, the abundances of 75 metabolites were significantly changed among all metabolites matched to the database (*p* < 0.05). These altered metabolites were further analyzed using one-way ANOVA, yielding 30 metabolites that were differentially regulated between the control, model, and CCFM1025 groups (Figure 3A and Supplementary Table S1). Notably, the levels of L-tyrosine and tryptophan in the model group were restored by *B. breve* CCFM1025 treatment, resulting in clustering of the CCFM1025 serum metabolomes of the control and model groups (Figure 2A).

### 3.3. B. breve CCFM1025 Supplementation Restored the Aβ-Induced Hippocampal Metabolome

As numerous metabolites can cross the blood–brain barrier (BBB), and cognitive impairment may result in increased permeability [18], we further utilized metabolomic profiling to examine metabolites in the hippocampus of control, model, and CCFM1025 mice. A total of 1549 metabolites, including amino acids, bile acids, carbohydrates, and lipids, were identified in the hippocampus. When compared with the control mice, 36 metabolites were statistically altered in the hippocampal tissues of the model mice (*p* < 0.05). Furthermore, using one-way ANOVA, we found a series of 19 altered metabolites that were differentially regulated between the control, model, and CCFM1025 groups. Consistent with findings from the serum metabolite profiles, the hippocampal metabolite profiles discriminated the Aβ-injected model group from the control group (Figure 3B and Supplementary Table S2). Intriguingly, the majority of the differentiated hippocampal metabolites are relevant to amino acid metabolism, including glutamic acid, anserine, taurine, and N-acetylaspartic acid.

### 3.4. Metabolic Alterations Were Identified in CCFM1025 Mice Compared with Model Mice

**Figure 4:**
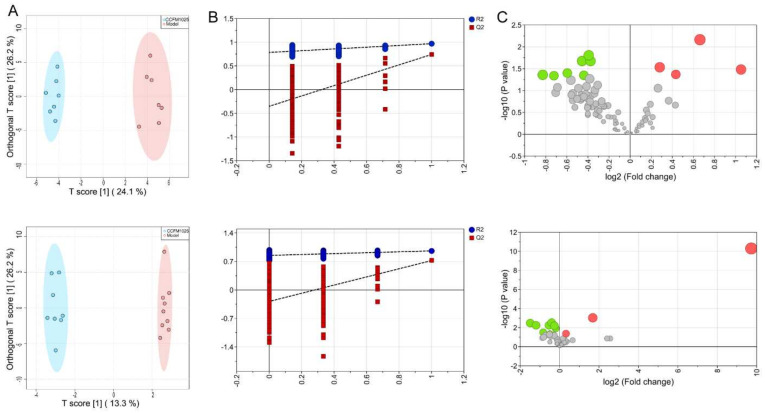
Metabolic alterations were identified in *B. breve* CCFM1025 compared with the model group in ESI^−^ mode. (**A**) OPLS-DA score plots for serum (up) and hippocampus (down) samples. The X and Y axis represent the contribution of the first two principal components (PC1 and PC2). (**B**) Cross-validation plot for the serum (up) and hippocampus (down) OPLS-DA model with a permutation test repeated 200 times. The intercepts of R2 = (0.0, 0.785) and Q2 = (0.0, –0.351) and R2 = (0.0, 0.849) and Q2 = (0.0, –0.283) suggest that the OPLS-DA model is not overfitting. (**C**) Volcano plots showing the results of pairwise comparisons of serum (up) and hippocampal (down) metabolites in the *B. breve* CCFM1025 and model group. Metabolites with significant changes are presented in red (upregulated) or green (downregulated). ESI^−^: negative electrospray ionization; OPLS-DA: orthogonal partial least squares discrimination analysis.

To further identify discriminatory features in the CCFM1025 group compared with the model group, we performed pairwise multivariate analysis. The OPLS-DA score plot demonstrated distinct clusters of metabolites in serum (*p* < 0.05) and hippocampus samples in both ESI^−^ (Figure 4A) and ESI^+^ models (Supplementary Figure S3A). The ability of OPLS-DA models to correctly discriminate the samples was checked by cross-validation through 200 permutation tests (Figure 4B). The values of goodness-of-fit (R2) and predictive ability (Q2) indicated that the OPLS-DA models were not overfitted.

**Figure 5:**
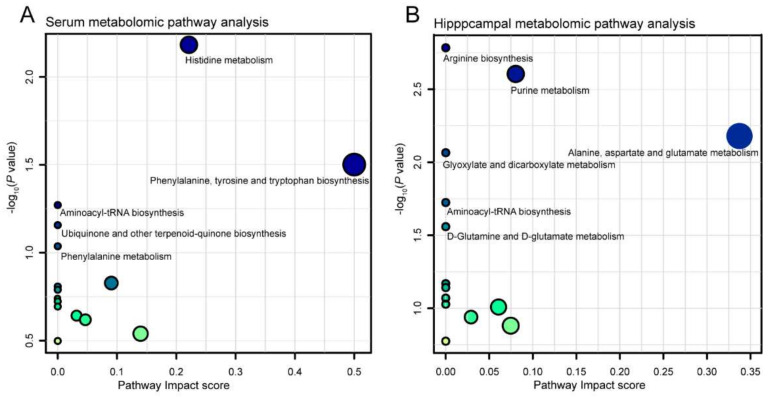
Scatterplot showing results of MetaboAnalyst Pathway analysis using the Mus musculus (mouse) pathway library. (**A**) Serum; (**B**) hippocampus.

Considering the VIP, FC, and *p* value, volcano plots were used to illustrate differential metabolites between the *B. breve* CCFM1025 and model groups in both the ESI^−^ (Figure 4C) and ESI^+^ models (Supplementary Figure S3C) of the serum and the hippocampus. Notably, 20 metabolites in the serum (Table 1) and 26 metabolites (Table 2) in the hippocampus exhibited significant changes between the model and the *B. breve* CCFM1025 groups. Functionally, these differentially changed metabolites were enriched in five serum metabolic pathways (Figure 5A) and six hippocampal metabolic pathways (Figure 5B), including histidine metabolism, phenylalanine metabolism, arginine biosynthesis, purine metabolism, and D-glutamine and D-glutamate metabolism, which are mainly linked to amino acid metabolism and aminoacyl transfer RNA biosynthesis

### 3.5. B. breve CCFM1025 Inhibited Aβ-Induced Neuroinflammation by Regulating Amino Acid Metabolism

**Figure 6:**
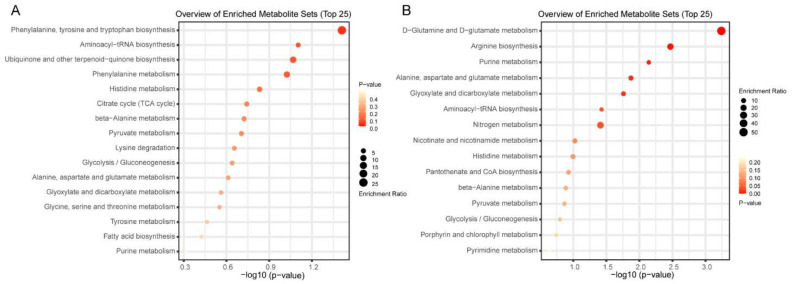
Metabolite set enrichment analysis of significantly altered metabolites in serum (**A**) and hippocampus (**B**) according to the KEGG database. Note: KEGG, Kyoto Encyclopedia of Genes and Genomes.

To further identify biologically meaningful patterns in *B. breve* CCFM1025 mice for the potential attenuation of AD, we performed metabolite set enrichment analysis (MSEA) using the KEGG database as a framework. As shown in Figure 6, the results revealed notable enrichment of several processes, including phenylalanine, tyrosine, and tryptophan biosynthesis and aminoacyl tRNA biosynthesis in serum, and arginine biosynthesis and D-glutamine and D-glutamate metabolism in the hippocampus.

**Figure 7:**
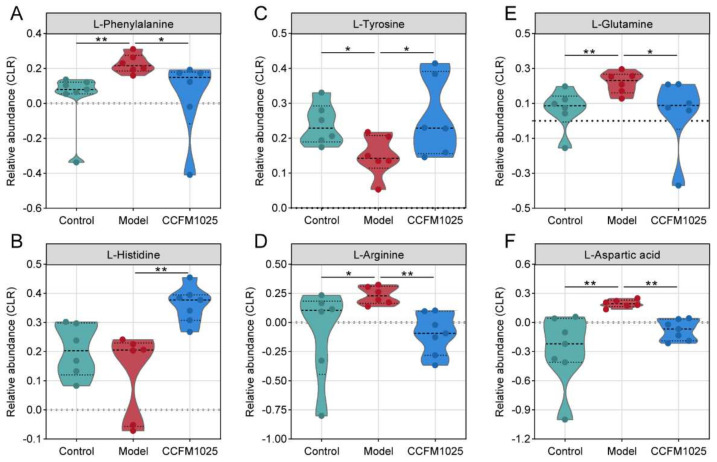
Violin plots representing the significantly altered metabolites after *B. breve* CCFM1025 treatment. Black horizontal lines in violin plots depict the medians. The *y* axis shows CLR-transformed metabolite concentrations. Mann–Whitney *U*-test (two-sided) post hoc tests, * *p*  <  0.05; ** *p*  <  0.01.

The enriched categories contained numerous essential metabolites, predominantly amino acids, which are crucial for cognition and proper brain function [19]. Specifically, CCFM1025 supplementation restored the level of the serum metabolite phenylalanine (Figure 7A), which plays critical roles in neurotransmission and aging [19]. Similar changes were found in L-glutamine levels in the hippocampus (Figure 7E). Moreover, we observed widespread increases in the levels of L-arginine in the serum and L-aspartic acid in the hippocampus in the model group compared with the control group (Figure 7D–F). The elevated levels of these metabolites were significantly reduced by supplementation with *B. breve* CCFM1025 (Figure 7D–F). Conversely, the levels of L-tyrosine and L-histidine were decreased in model mice and statistically reversed by *B. breve* CCFM1025 treatment.

## 4. Discussion

In this study, we performed untargeted metabolomic analysis of serum and hippocampus samples to identify AD-associated metabolic alterations and determine the effect of a psychobiotic on the progression of AD. Here, we demonstrated that Aβ injection altered the metabolomic composition in a mouse model, and changes in the metabolites were partially reversed by a *B. breve* CCFM1025 intervention. Among the differentially altered metabolites, phenylalanine is crucial for cognition and brain plasticity, and L-glutamine plays a protective role in neuroinflammation. Given that selected metabolites can cross the BBB and enable brain–gut axis crosstalk, we propose that the *B. breve* CCFM1025 intervention promotes neuroprotective metabolites in the gut that may be transported into the brain to alleviate neuroinflammation and influence brain function.

Metabolites can be detected in multiple biological samples, and it is apparent that they exert profound and diverse effects on host health [9,20]. The dramatic alteration of serum metabolites in AD has been illustrated by comparing APP/PS1 mice with wild-type control mice, including changes in the relative proportions of amino acids and a lack of fatty acids [21]. Similar findings were also observed in a human study that compared the serum metabolites of healthy individuals and AD patients, illustrating the importance of amino acids in a complex metabolome [13]. Consistent with those findings, significant alterations in the serum metabolite profiles in model mice relative to those of control mice were seen in our study. In addition to serum metabolomes, mouse model studies have described differences in hippocampal metabolites between control and AD mice, including a lack of N-glycolylneuraminate in AD mice, and several hippocampal metabolites have been closely linked to amino acid metabolism, which is crucial for neurotransmission and aging [19,22]. The results from our study also revealed changes in numerous amino acids in the model mice, though the types of amino acids are not exactly the same due to the different experimental mouse models used. Together, our findings collectively demonstrate that the compositions of metabolites in the serum and hippocampus were altered during AD progression, with amino acids as a potential driver.

Given our previous findings that *B. breve* CCFM1025 can modulate the gut microbiota composition and that Aβ-induced cognitive impairment can be ameliorated by dietary supplementation, which helps to improve brain function [6], the modulation of key metabolites may explain the brain benefits of *B. breve* CCFM1025. One published study demonstrated that a lack of gut microbiota is associated with increased BBB permeability in both the fetal and adult mouse brain. Strikingly, treatment of germ-free mice with bacteria that produce metabolites decreased the permeability of the BBB and in so doing allows proper functioning of neurons [23]. In addition, a large proportion of gut-derived metabolites can cross the BBB, including N-glycolylneuraminate, which is closely linked to microglial phagocytosis and neuroinflammation [24], and arginine, which is correlated with neurodegeneration and the nitric oxide pathway in AD [25].

As the BBB is disrupted when cognitive declines occur in neurodegenerative diseases, the increased permeability may strengthen communication between the blood and the brain [26]. Through the gut–brain axis, gut-derived metabolites are transported into the brain and therefore influence brain function. For instance, one serum metabolomic study showed that gut microbiota-derived metabolites were altered in patients with early stage Huntington’s disease [27]. Sodium oligomannate suppressed gut bacterial amino acids and inhibited neuroinflammation in AD progression [28]. In a chronic stress-induced mouse model, *B. breve* CCFM1025 produced neuro-modulatory metabolites, including hypoxanthine, tryptophan, and nicotinate [29]. In addition, a *Lactobacillus* intervention increased the gamma-aminobutyric acid (GABA), N-acetylaspartate, and glutamate levels in the hippocampus as detected by magnetic resonance spectroscopy [30]. As such, *B. breve* CCFM1025 may modulate the serum and hippocampal metabolomes through indirect or direct mechanisms in the early stage of AD, as several of the differential metabolites can cross the BBB.

Amino acids are transported across the BBB [31]. Serving as nitrogen donors and neurotransmitters, amino acids, mostly L-glutamine, play critical roles in the central nervous system [32]. Through further pathway and enrichment analysis, we identified that the amino acid metabolism pathway and aminoacyl tRNA biosynthesis play important roles in AD progression, which aligns with previous studies linking amino acid metabolism with aging and cognitive decline [28,33,34]. The observed modulation in amino acids, such as L-glutamine and L-arginine, suggest that chronic oral *B. breve* CCFM1025 treatment likely remodels amino acid metabolism. Notably, arginine is linked to the nitric oxide pathway and neurodegeneration [25]; however, a *B. breve* CCFM1025 intervention may cause a reversal in arginine levels. The level of L-glutamine, a precursor of glutamate and GABA, was reported to be altered in the hippocampus of a mouse model of seizure and has been implicated in behavior responses [15]. In line with previous research, we found that changes in hippocampal L-glutamine levels in model mice were reversed by *B. breve* CCFM1025 supplementation. Overall, these findings indicated that the levels of these metabolites can be restored by *B. breve* CCFM1025 treatment and therefore influence brain function, and that amino acids may drive this process.

We acknowledge that our study has some limitations. Firstly, we only performed untargeted metabolomics to identify the differential metabolites. Further targeted metabolomic analysis is necessary to increase the rigor and confidence of our findings. While our study examined metabolic profiles in the serum and the hippocampus, a future investigation is warranted to explore whether the fecal metabolome is also linked to amino acid metabolism. As we focused mainly on metabolomic data, longitudinal multi-omics data in future studies could help to understand the mechanisms behind the protective effects of *B. breve* CCFM1025 on brain function in the development of AD.

## 5. Conclusions

Overall, our study revealed that *B. breve* CCFM1025 supplementation could modulate the serum and hippocampal metabolomes in Aβ-injected AD model mice. Further pathway analysis and MSEA revealed that the metabolites were predominantly related to amino acid metabolism, which is crucial for cognition and proper brain health. While these findings lend credence to future research determining the impact of psychobiotic interventions on metabolite alterations in AD progression, several additional studies are needed to explore whether metabolite-based treatments can be effectively applied for the amelioration of cognitive impairment.

## Figures and Tables

**Table 1:** The significantly altered metabolites in serum between the CCFM1025 and model groups.

Metab_id	Metabolite	OPLS-DA VIP	*p* Value	Fold Change	HMDB ID	KEGG ID
N173	Plumbagin	2.09326	0.00678447	1.58203	HMDB0035291	-
N232	Ascorbicacid-2-sulfate	1.86579	0.0157301	0.761099	-	-
N187	Decanoic acid	1.82526	0.0209732	0.770978	HMDB0000511	C01571
N96	No-noic acid	1.84173	0.021134	0.729044	HMDB0000847	C01601
N219	L-Tyrosine	1.70911	0.0292742	1.21693	HMDB0000158	C00082
N289	2-(2-Acetoxy-2-oxoethyl)-2-hydroxysuccinic acid	1.71639	0.0326245	2.0757	-	-
N271	5-[(2Z,8Z)-2,8-Pentadecadien-1-yl]-1,3-benzenediol	1.64572	0.0396629	0.662185	-	-
N6	L-(+)-Lactic acid	1.6948	0.0423889	1.35085	HMDB0000190	C00186
N55	Citric acid	1.7116	0.0433744	0.563898	HMDB0000094	C00158
N98	Cytidine;1-beta-delta-Ribofuranosyl-Cytosine	1.59184	0.0450476	0.738744	-	-
N135	[FA(18:4)]6Z_9Z_12Z_15Z-octadecatetraenoicacid	1.6114	0.046132	0.605545	-	-
P93	Hexanoylglycine	2.19498	0.00415879	0.57031	HMDB0000701	-
P132	(4R)-5-Hydroxy-L-leucine	2.13034	0.00443283	0.766452	HMDB0000450	C16741
P15	Betaine	1.99501	0.00767035	1.43377	HMDB0000043	C00719
P95	L-Histidine	1.88292	0.0125969	1.42264	HMDB0000177	C00135
P280	Capsidiol	1.90628	0.0165168	0.627435	HMDB0002352	C09627
P209	4121	1.90749	0.0171203	0.579715	HMDB0000462	C01551
P55	N,N-Dimethyldecylamine N-oxide	1.8191	0.0239789	1.96321	HMDB0001466	C01183
P189	1-Methylhistidine	1.74198	0.0325697	0.846794	HMDB0000001	C01152
P113	2475675	1.69582	0.0366034	0.556372	HMDB0015593	-

**Table 2:** The significantly altered metabolites in the hippocampus between the CCFM1025 and model group.

Metab_id	Metabolite	OPLS-DAVIP	*p* Value	Foldchange	HMDB ID	KEGGID
N80	Abieticacid	3.69022	4.98E-11	845.997	HMDB0000042	C00033
N92	Hypoxanthine	2.82776	0.00092	3.21625	HMDB0000157	C00262
N74	1-Stearoyl-2-hydroxy-sn-glycero-3-PE	2.52581	0.003066	0.751745	-	-
N239	2-(2-Carboxyethyl)-4-methyl-5-pentyl-3-furoic acid	2.48185	0.00567	0.435581	-	-
N85	D-(-)-Glutamine	2.47921	0.00346	0.352861	HMDB0000641	C00064
N23	2,4-di-tert-Butylphenol	2.4217	0.005509	0.685152	HMDB0013816	-
N292	1-Palmitoyl-2-hydroxy-sn-glycero-3-PE	2.34582	0.006262	0.834934	-	-
N14	L-Aspartic acid	2.21739	0.011711	0.867782	HMDB0000191	C00049
N3	Dodecyl sulfate	2.0113	0.031644	0.562855	-	-
N238	Cytidine;1-beta-delta-Ribofuranosyl-Cytosine	1.96217	0.040588	1.23977	-	-
N16	Myristyl sulfate	1.90313	0.042206	0.704926	-	-
P1146	Diethyl phthalate	1.92305	0.046523	0.624987	-	-
P743	Dichloromethane	1.81003	0.044936	1.67818	HMDB0031548	C02271
P145	Adenosine 5’-monophosphate	2.00506	0.041507	1.67312	HMDB0000045	C00020
P215	1-Oleoyl-2-hydroxy-sn-glycero-3-PE	1.86473	0.041022	1.50993	-	-
P628	PAF C-18:1	1.9106	0.039774	1.54938	-	-
P1068	methyl 2,8-dihydroxy-6-(hydroxymethyl)-9-oxo-2,9-dihydro-1H-xanthene-1-carboxylate	1.89251	0.039566	1.45483	-	-
P109	Adenosine 5’-monophosphate	2.00566	0.034384	1.63448		
P1049	Docosahexaenoyl Ethanolamide	1.93602	0.032247	1.547	HMDB0013658	-
P144	Sangivamycin	2.03425	0.031676	1.63017	-	-
P912	11-Nitro-1-undecene	2.05666	0.025121	1.76411	-	-
P1156	4049	2.05316	0.02406	1.65415		
P489	Biliverdin	1.97892	0.023273	7.11057	HMDB0015624	-
P1071	mebutamate	2.19096	0.018291	1.56646	HMDB0001008	C00500
P800	1-Stearoyl-2-hydroxy-sn-glycero-3-PE	2.12975	0.017473	1.70019	-	-
P393	1-stearoyl-lysophosphatidylcholine	2.24008	0.012303	1.67575	-	-

## Data Availability

The data that support the findings of this study are available on request from the corresponding author.

## Supplementary Materials

The following supporting information can be downloaded at: https://www.mdpi.com/article/10.3390/nu14040735/s1, Figure S1: The flow chart of processing workflow used in this study; Figure S2: Quality control assessment; Figure S3: Metabolic alterations were identified in *B. breve* CCFM1025 compared with model group in ESI^+^ mode; Tables S1 and S2: The differential metabolites in serum and hippocampus among three groups using one-way ANOVA.
