# Supplementary material for: Integrative Metabolomic Characterization Reveals the Mediating Effect of Bifidobacterium breve on Amino Acid Metabolism in a Mouse Model of Alzheimer’s Disease
Source: Nutrients. 2022 Feb 9;14(4):735. doi: 10.3390/nu14040735 (PMC8878368; doi:10.3390/nu14040735)
Supplement: Supplementary file 1 [file nutrients-14-00735-s001.zip › nutrients-1569418-supplementary.pdf]

Supplementary Tables

Table S1. The differential metabolites in serum among three groups using one-way ANOVA.

Metab_id	Metabolite	Chi squared	P value
P158	LysoPC(22:5(7Z,10Z,13Z,16Z,19Z))	16.154	0.000311
P95	LysoPC(22:5(7Z,10Z,13Z,16Z,19Z))	14.672	0.000652
N219	L-Tyrosine	14.514	0.000705
N206	(2S)-N2-[(2S)-1-amino-3-cyclohexyl-1-oxo-2-propanyl]-N1-isopropyl-N4-phenyl-1,2,4-piperazinetricarboxamide	14.417	0.00074
P106	L-alpha-lysophosphatidylcholine	14.416	0.000741
N223	L-Tyrosine	13.668	0.001077
N20	cis-5,8,11,14,17-Eicosapentaenoic acid	12.455	0.001974
N67	L-Phenylalanine	12.145	0.002305
P24	Dibutyl phthalate	11.634	0.002976
N297	2-Hydroxymyristic acid	10.76	0.004608
N51	D-(+)-Tryptophan	10.756	0.004617
P52	LysoPC(20:5(5Z,8Z,11Z,14Z,17Z))	10.314	0.005758
P70	Docosahexaenoic acid	10.272	0.005882
P15	Betaine	10.189	0.006129
P306	Methyl palmitate	10.143	0.006274
P11	DA9185000	10.137	0.006292
N271	5-[(2Z,8Z)-2,8-Pentadecadien-1-yl]-1,3-benzenediol	10.01	0.006703
N236	LT9970000	9.8739	0.007176
N173	Plumbagin	9.8482	0.007269
N257	(R)-10-Hydroxystearate	9.7781	0.007529
N71	Adrenic acid	9.7746	0.007542
N94	3-Oxotetradecanoic acid	9.5713	0.008349
N5	Docosahexaenoic Acid	9.5537	0.008423
P10	2-Amino-1,3-octadecanediol	9.1746	0.01018
N266	Bufanolide	8.8914	0.011729
N274	3-(3,4-Dimethyl-5-pentyl-2-furyl)propanoic acid	8.7981	0.012289
P55	N,N-Dimethyldecylamine N-oxide	8.7482	0.012599
N172	Tridecylic acid	8.6717	0.01309
P7	1-phenylpropane-1_2-dione	8.5282	0.014064
P8	L-Phenylalanine	8.5282	0.014064

Table S2. The differential metabolites in hippocampus among three groups using one-way ANOVA.

Metab_id	Metabolite	Chi squared	P value
N80	Abieticacid	2037.6	1.30E-26
N56	6-Aminocaproic acid	249.16	2.59E-16
N66	Glycerol 3-phosphate	19.556	1.09E-05
N165	Hydroxymethylphosphonate	18.047	1.94E-05
N89	Deoxyguanosine diphosphate (dGDP)	14.927	6.99E-05
N110	Guanosine monophosphate (GMP)	11.998	0.00027
N18	L-Glutathione oxidized	11.987	0.000271

N93	UDP-N-acetylglucosamine	11.901	0.000283
N292	1-Palmitoyl-2-hydroxy-sn-glycero-3-PE	11.553	0.000336
N4	L-Glutamic acid	11.253	0.000391
N117	Uridine 5'-diphosphogalactose	11.16	0.00041
N195	Anserine	11.142	0.000413
N286	D-Erythrose4-phosphate	11.031	0.000438
N143	butanethiol	10.91	0.000466
N127	Inosine-5'-monophosphate (IMP)	10.472	0.000584
N238	Cytidine;1-beta-delta-Ribofuranosyl-Cytosine	9.7704	0.000848
N5	Taurine	9.4425	0.001014
N30	Adenine	8.5595	0.001665
N2	N-Acetylaspartic acid	8.5434	0.00168

Supplementary Figures and Figure legends

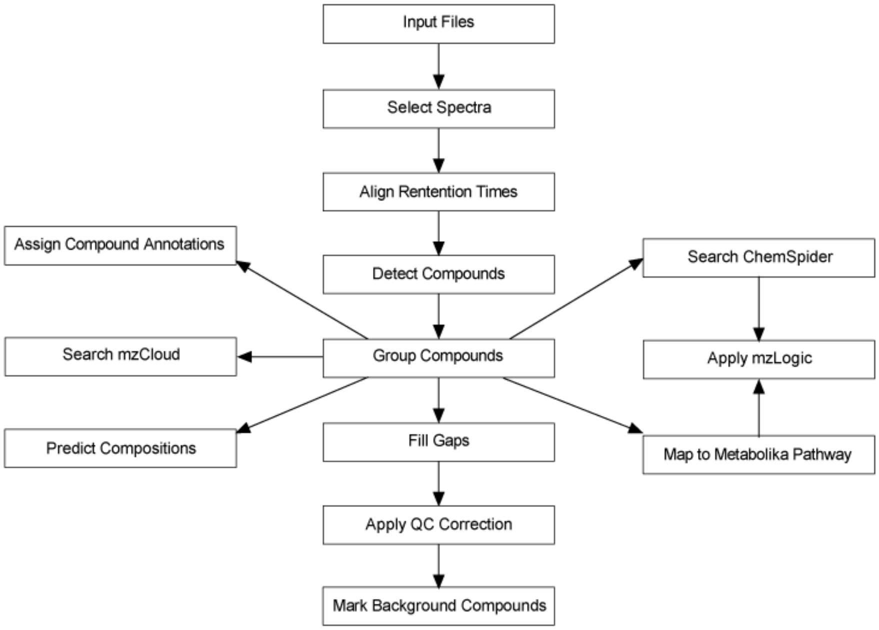

Figure S1. The flow chart of processing workflow used in this study.

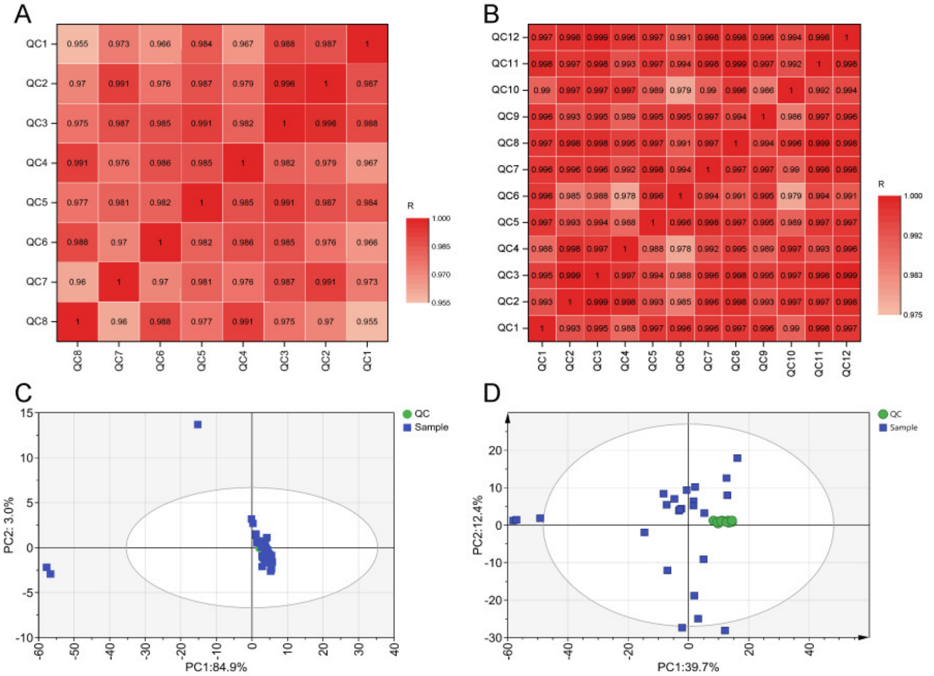

Figure S2. Quality control assessment. The Pearson correlation of serum (A) and hippocampus tissues (B) in ESI$^+$ mode. The PCA score plots for all serum (C) and hippocampus (D) samples containing QC samples in ESI$^+$ mode. Note: ESI$^+$, negative electrospray ionization.

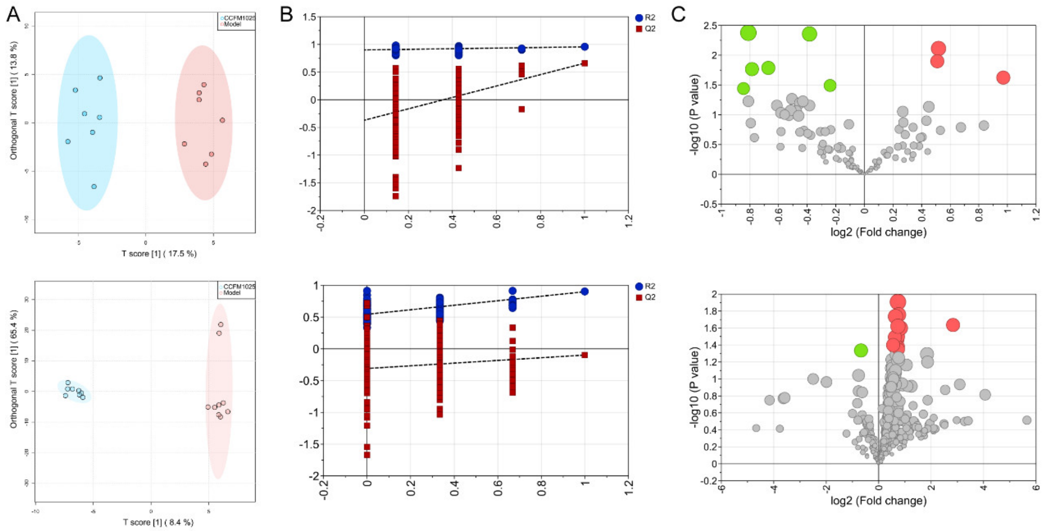

Figure S3. Metabolic alterations were identified in *B. breve* CCFM1025 compared with model group in ESI$^+$ mode. (A) OPLS-DA score plots for serum (up) and hippocampus (down) samples. X and Y axis represent the contribution of the first two principal components (PC1 and PC2). (B) Cross-validation plot for serum (up) and hippocampus (down) OPLS-DA model with a permutation test repeated 200 times. The intercepts of R2= (0.0, 0.9) and Q2= (0.0, –0.37) and R2= (0.0, 0.544) and Q2= (0.0, –0.309) suggest that the OPLS-DA model is not overfitting. (C) Volcano plots showing the results of pairwise comparisons of serum (up) and hippocampal (down) metabolites in *B. breve* CCFM1025 and model group. Metabolites with significant changes are presented in red (upregulated) or green (downregulated). Note: ESI$^+$, positive electrospray ionization; OPLS-DA, Orthogonal partial least squares discrimination analysis.
